# Diagnostic issues and capabilities in 48 isolation facilities in 16 European countries: data from EuroNHID surveys

**DOI:** 10.1186/1756-0500-5-527

**Published:** 2012-09-25

**Authors:** Simon-Djamel Thiberville, Stefan Schilling, Giuseppina De Iaco, Francesco Maria Fusco, Gail Thomson, Helen C Maltezou, Rene Gottschalk, Reinhard H Brodt, Barbara Bannister, Vincenzo Puro, Giuseppe Ippolito, Philippe Brouqui

**Affiliations:** 1 UMR 190, Emergence of Viral Pathologies, Aix-Marseille Univ-IRD-EHESP French School of Public Health, Marseille, France; 2 Infectious Disease and Tropical Medicine, AP-HM CHU Nord, University Hospital Institute for Infectious Disease and Tropical Medicine, Marseille, France; 3 Department for Infectious Diseases, Goethe University Hospital, Frankfurt, Germany; 4 National Institute for Infectious Diseases "L. Spallanzani", Epidemiology and Pre-clinical Research Department, Rome, Italy; 5 Health Protection Agency, Porton Down, Salisbury, UK; 6 Department for Interventions in Health-Care Facilities, Hellenic Center for Disease Control and Prevention, Athens, Greece; 7 Health Protection Authority, Frankfurt am Main, Germany; 8 Royal Free Hospital, London, UK

**Keywords:** Diagnostic techniques and procedures, European Union, Communicable diseases, Infection control, Patient isolation, Critical pathway

## Abstract

**Background:**

Highly infectious diseases (HIDs) are defined as being transmissible from person to person, causing life-threatening illnesses and presenting a serious public health hazard. The sampling, handling and transport of specimens from patients with HIDs present specific bio-safety concerns.

**Findings:**

The European Network for HID project aimed to record, in a cross-sectional study, the infection control capabilities of referral centers for HIDs across Europe and assesses the level of achievement to previously published guidelines. In this paper, we report the current diagnostic capabilities and bio-safety measures applied to diagnostic procedures in these referral centers. Overall, 48 isolation facilities in 16 European countries were evaluated. Although 81% of these referral centers are located near a biosafety level 3 laboratory, 11% and 31% of them still performed their microbiological and routine diagnostic analyses, respectively, without bio-safety measures.

**Conclusions:**

The discrepancies among the referral centers surveyed between the level of practices and the European Network of Infectious Diseases (EUNID) recommendations have multiple reasons of which the interest of the individuals in charge and the investment they put in preparedness to emerging outbreaks. Despite the fact that the less prepared centers can improve by just updating their practice and policies any support to help them to achieve an acceptable level of biosecurity is welcome.

## Findings

### Background

A highly infectious disease (HID), such as severe acute respiratory syndrome (SARS) [1], Lassa fever [2], or other hemorrhagic fever [3,4], is defined as transmissible from person to person, causes a life-threatening illness, presents a serious hazard in healthcare settings and in the community and requires specific control measures [5]. Suspected HID patients should be managed in specialized isolation facilities, such as “high-level isolation units” [5].

To prepare the hospital management of HIDs for possible future outbreaks, the European Union (EU) recently funded the European Network of Infectious Diseases (EUNID) [5]. Following EUNID, the European Network for Highly Infectious Diseases (EuroNHID), a new EU-funded project, performed a cross-sectional study analysis of European isolation facilities. The specific mission of EuroNHID is to prepare and support isolation facilities to provide appropriate infection control measures and strategies for health care worker (HCW) safety during care to patients with suspected and confirmed HIDs.

The appropriate management of HID cases requires high-level diagnostic capabilities.

In 2009, the EUNID has reached a consensus on recommended biosafety procedures for the entire diagnostic process, from specimen sampling to the transport of laboratories [6]. The aim of this paper is to report the current inventory of the diagnostic capabilities and infection control procedures for the appropriate and safe handling of specimens in 48 isolation facilities in 16 European countries who participated to the cross-sectional study of the EuroNHID.

### Methods

#### Settings and participants

At the beginning of the project, national health authorities in all of the European countries were asked to suggest a physician with expertise in HID management as a project partner and to identify all the isolation facilities as referral centers for HID in his country. To survey only isolation facilities identified by national health authorities for the referral and management of HIDs, we requested official documents in which these hospitals are clearly indicated.

#### Data collection

The data were collected during on-site visits using checklists specifically developed during the first year of the project [7]. The checklists were drafted by the steering committee members (including partners from France, Germany, Greece and the United-Kingdom) and then discussed with and approved by all of the partners. The coordination team (based at the National Institute for Infectious Diseases in Rome, Italy) considered the strength scores of these assessments to be indispensable.

On-site visits were performed by the project coordinator assisted, when feasible, by the project partner of the explored country from February-November 2009.

#### Objectives and data analysis

The objective of the project was to assess the level of achievement of each surveyed facility to previously published guidelines [5,6]. With this aim, a standard evaluation form was developed. In this form, all of the data were summarized in main topics, and for each topic, an evaluation score was assigned that represented the level of achievement with respect to a “standard” based on the available evidence, literature data and expert consensus [5,6].

The checklists and the evaluation form are available on the website http://www.eunid.eu after free registration.

### Results

The participant selection process led to the inclusion of 48 isolation facilities identified for the referral and management of HIDs in 16 countries Table 1.

**Table 1:** Diagnostic capabilities, appropriate location and procedures for microbiological and routine tests in 48 referral center for HID in 16 European countries

**Diagnostic capabilities**	Evaluation score*		
	**A Fully/mostly**	**B Partially**	**C Not**
	**Achieved**	**achieved**	**Achieved**
	**N (%)**	**N (%)**	**N (%)**
Topic 1: The isolation facility has access to BSL-4 labs or capabilities/protocols for the safe and appropriate handling of group 4 agent specimens for diagnosis	11/48 (23)	30/48 (62)	7/48 (15)
Topic 2: The isolation facility has access to BSL-3 labs or capabilities/protocols for the safe and appropriate handling of group 3 agent specimens for diagnosis	39/48 (81)	9/48 (19)	0/48
Topic 3: The isolation facility has capabilities/procedures for the safe and appropriate management of other tests/routine analysis in HID patients (i.e., use of bed-side tests inside isolation area or use of central hospital lab after inactivation of samples or without inactivation but with appropriate measures of biosecurity and biosafety, including the use of automatic, closed-type system analyzers)	16/48 (33)	19/48 (40)	13/48 (27)
**Appropriate location and procedures for microbiological or routine tests**	**microbiological test**	**routine test**	
	**N (%)**	**N (%)**	
Inside the isolation area (same room or other room)	8/47 (17)	13/48 (27)	
In the BSL-3 reference lab	32/47 (68)	15/48 (31)	
In the general lab, with closed-type automatized analyzers	19/47 (40)	26/48 (54)	
In the general lab, without closed-type automatized analyzers	5/47 (11)	15/48 (31)	

*The corresponding assessment of the evaluation scores of the three topics are summarized below:

**Topic 1: management of group 4 agent specimens** (**A**: The unit is located in the same hospital/city as a BSL-4 lab and has protocols for the safe and appropriate handling of group 4 agent specimens; **B**: The unit is not located in the same hospital/city as a BSL-4 lab but has protocols for the safe and appropriate handling of group 4 agent specimens to another city/country; **C**: The unit is not located in the same hospital/city as a BSL-4 lab and does not have adequate protocols for the safe and appropriate handling of group 4 agent specimens to another city/country). **Topic 2: management of group 3 agent specimens** (**A**: The unit is located in the same hospital/city as a BSL-3 lab and has adequate protocols for the safe and appropriate handling of group 3 agent specimens; **B**: The unit is not located in the same hospital/city as a BSL-3 lab but has adequate protocols for the safe and appropriate handling of group 3 agent specimens to another city; **C**: The unit is not located in the same hospital/city as a BSL-3 lab and does not have adequate protocols for the safe and appropriate handling of group 3 agent specimens to another city). **Topic 3: management of other tests/routine analysis** (**A**: Optimal use of bed-side testing inside the isolation area OR use of the central hospital lab after inactivation of samples OR use of the BSL-3 lab; **B**: Use of the central hospital lab without inactivation with special measures of biosecurity and biosafety, including the use of automatic, closed-type system analyzers; **C**: Use of central hospital lab without special measures of biosecurity and biosafety).

Throughout Europe, 26 (54%) of the surveyed isolation facilities have a biosafety level 4 (BSL-4) laboratory (lab) [8] in their country, 11 of which are located in the same hospital or city as the isolation facility to avoid or limit the distance of the transportation of HID diagnostic specimens. Among those isolation facilities not located in the same city as the BSL-4 lab, 30 (62%) have written protocols for the appropriate handling and transportation of the specimens to a BSL-4 lab, and 7 (15%) have no specific protocols.

Among the isolation facilities surveyed, 48 (100%) and 47 (98%) have a BSL-3 lab [8] for virological and bacteriological diagnosis in their country, respectively. In total, 39 (81%) of the isolation facilities are located in the same hospital or city as the BSL-3 lab, and all of the isolation facilities have an adequate protocol for the safe and appropriate handling of group 3 agents.

Sixteen (33%) isolation facilities have access to adequate capabilities for other routine diagnostic tests: (i) optimal use of bed-side testing inside the isolation area, (ii) use of the central hospital lab after the inactivation of samples, and (iii) use of the BSL-3 lab [6]. However, 19 (40%) of the isolation facilities perform routine analysis (such as biochemistry and hematology) in the central hospital lab without inactivation but using special measures of biosecurity and biosafety, such as closed-type auto-analyzers. The remaining 13 (27%) facilities perform other diagnostic tests in the central laboratory without any special measures of biosecurity and biosafety.

Microbiological and routine diagnostic tests are performed directly inside the isolation area in 8 (17%) and 13 (27%) of the surveyed facilities, respectively. Microbiological testing in the majority (32; 68%) of isolation facilities and routine testing in a small proportion (15; 31%) of the facilities are carried out in a BSL-3 lab. For 19 (40%) and 26 (54%) of the centers, the samples of microbiological and routine testing are sent to the central laboratory, which performs the analysis in a closed-type automatic analyzer. Finally, microbiological and routine tests are performed in the central laboratory without using closed-type auto-analyzers in 5 (11%) and 15 (31%) of the surveyed facilities, respectively.

### Conclusion

Although most of the isolation facilities surveyed have appropriate diagnostic capabilities and infection control procedures for the safe handling of specimens, 31% and 11% performed their routine and microbiological diagnostic tests in the central laboratory without any measures of biosecurity and biosafety as recommended by the EUNID [6]. The delay between data collection, and publication, can be considered as one of the limit of this paper.

The discrepancies among the referral centers surveyed between the level of practices and the EUNID recommendations have multiple reasons. One main explanation is the interest of the individuals in charge and the investment they put in preparedness to emerging outbreaks. Obviously, these centers might benefit from larger funding from their national institution, or if they better allocated their internal resources. Despite the fact that the less prepared centers can improve by just updating their practice and policies any support to help them to achieve an acceptable level of biosecurity is welcome.

## Abbreviations

HID: Highly infectious disease; EU: European Union; EUNID: European Network of Infectious Diseases; EuroNHID: European Network for Highly Infectious Diseases; HCW: Health Care Worker; BSL-4: Biosafety level 4; BSL-3: Biosafety level 3.

## Competing interests

The authors declare that they have no competing interests.

## Authors’ contributions

SDT and PB wrote the manuscript, SS, GDI, GT, HCM, RG, HRB, BB, VP, and PB substantial contributed to design the study, participated in the acquisition and interpretation of data, and gave the final approval of the version to be published; GI substantial contributed to design the study, and gave the final approval of the version to be published. All authors read and approved the final manuscript.
